# Incidence Rate and Risk Factors for Developing Active Tuberculosis Among People Living With HIV in Georgia 2019–2020 Cohort

**DOI:** 10.1093/ofid/ofae466

**Published:** 2024-08-19

**Authors:** Mariana Buziashvili, Mamuka Djibuti, Nestani Tukvadze, Jack DeHovitz, Davit Baliashvili

**Affiliations:** Scientific Research Unit, National Center for Tuberculosis and Lung Diseases, Tbilisi, Georgia; Department of Medicine, Tbilisi State University, Tbilisi, Georgia; Partnership for Research and Action for Health (PRAH), Tbilisi, Georgia; Scientific Research Unit, National Center for Tuberculosis and Lung Diseases, Tbilisi, Georgia; Department of Medicine, Swiss Tropical and Public Health Institute, Allschwil, Switzerland; Department of Medicine, State University of New York Downstate Health Sciences University, Brooklyn, New York, USA; Partnership for Research and Action for Health (PRAH), Tbilisi, Georgia

**Keywords:** HIV, incidence rate, people living with HIV, risk factors, tuberculosis

## Abstract

**Background:**

Tuberculosis (TB) is a leading cause of morbidity and mortality among people with HIV (PHIV) globally. Our study is the first to evaluate TB incidence and its risk factors among PHIV in the country of Georgia, where previously no data were available.

**Methods:**

A retrospective cohort study was conducted among persons newly diagnosed with HIV in Georgia during 2019–2020. Active TB incidence was calculated within a minimum of 2-year follow-up period from HIV diagnosis. Cox proportional hazard model was used for evaluating risk factors for TB development.

**Results:**

The median age in the final cohort of 1165 PHIV was 38 (interquartile range, 30–48) and 76.3% were male. Twenty-nine percent of patients had a CD4 cell count <200 at HIV diagnosis and 89.9% initiated antiretroviral therapy (ART). TB incidence rate was 10/1000 person-years (p-y; 95% confidence interval [CI], 9.6–10.4), with rates being higher within several subgroups, mainly: PHIV aged 40–49 years (17.5/1 000 p-y [95% CI, 16.8–18.2]); those not receiving ART (22/1000 p-y [95% CI, 20.9–23.1]); those with CD4 < 200 at baseline (28/1000 p-y [95% CI, 27.4–28.6]); and those who developed AIDS (29.1/1 000 p-y [95% CI, 28.6–29.6]). Age (aHR, 1.2; 95% CI, 1.03–1.39; *P* = .01) and AIDS diagnosis (aHR, 3.2; 95% CI, 3.06–27.9; *P* = .001) were associated with TB development, whereas high CD4 count was protective against TB (aHR, 0.18; 95% CI, .06–.61; *P* = .005).

**Conclusions:**

Study results highlight an imperative role of CD4 cell count management and the need for early HIV diagnosis and timely initiation of ART to ensure an effective immune response against tuberculosis, stressing the need for further in-depth evaluation of the TB preventive treatment delivery system's efficiency and gaps.

## Teaser

The study highlights high TB incidence rate and evaluates its risk factors among Georgian PHIV, emphasizing the underuse of preventive treatments and the necessity for integrated HIV/TB care, including early TB preventive treatment and ART administration, to mitigate TB-related morbidity and mortality.

## BACKGROUND

Human immunodeficiency virus/acquired immunodeficiency syndrome (HIV/AIDS) and tuberculosis (TB) stand as significant global health challenges, exerting a substantial impact on morbidity and mortality rates worldwide [1]. Despite considerable advancements in awareness, education, and availability of preventive measures, there were 1.7 million people newly infected with HIV in 2022 and 630 000 HIV-related deaths [1]. TB is a leading cause of morbidity and mortality among people with HIV (PHIV). Among an estimated 10.6 million new cases of TB in 2022, an estimated 6.3% were among PHIV [2] and an estimated 1.1 million died of TB, including 167 000 among PHIV [3].

HIV infection is the most commonly known risk factor for *Mycobacterium tuberculosis* (MTB) infection and development of active disease [4] because of its severe impact on the host cell-mediated response to MTB, particularly through the depletion of CD4+ T cells [5, 6]. As a result, the risk of developing active TB increases 2- to 3-fold within the first 2 years of HIV seroconversion [7]. PHIV have an estimated 18 (15–21) times higher risk of developing active TB disease compared to those without HIV [8] and face a 5%–10% annual risk of latent TB reactivation [9, 10]. Antiretroviral therapy (ART) effectively replenishes depleted CD4 cells, thereby reducing TB risk by 67% and halving TB recurrence rates [11–14], together with the World Health Organization (WHO) recommended TB preventive treatment (TPT) [15, 16]. Globally in 2022, only 76% (65%–89%) of all PHIV were accessing antiretroviral treatment [1] and of HIV-positive TB patients with known HIV status (7.3%), 89% were receiving ART [2].

Although HIV is a well-established risk factor for TB, the incidence of TB among PHIV varies significantly by setting and there are limited data from cohort studies in Eastern Europe. Georgia is a country with increasing tendency in HIV incidence. In 2022, there were 16.5 incident cases per 100 000 population. Totally, there were an estimated 9779 cases of PHIV in Georgia at the end of 2022. Despite universal access to HIV services in Georgia, only 72% (n = 6050) of PHIV received ART as of 2022 [17]. Similar to global statistics, TB remains a leading cause of death among PHIV in Georgia, accounting for 21% of all deaths [1, 18, 19]. The incidence of TB in Georgia in 2022 equaled 60 per 100 000 population, with HIV incidence among people with new TB diagnoses being 1.7 cases per 100 000 population [20]. HIV prevalence among all TB patients in 2022 was 3.1%, although data on TB incidence among PHIV in Georgia are extremely limited. Despite a decline, drug-resistant tuberculosis continues to pose a significant challenge in Georgia. In 2022, rifampicin resistance was detected in 10.7% of newly diagnosed TB cases and 23.6% of retreatment cases, with 6% prevalence of HIV among confirmed drug-resistant TB cases in the same year.

To our knowledge, there are only few studies evaluating active TB disease among persons newly diagnosed with HIV [21, 22], but no further data are available on TB incidence and related risk factors among PHIV in Georgia; thus, our study aimed to evaluate the incidence and risk factors for active TB development among people newly diagnosed with HIV in Georgia. The specific objectives of the study were to: (1) determine the rate of TB incidence among PHIV in Georgia, who were newly diagnosed with HIV during the years 2019–2020; (2) assess average time to TB development from HIV diagnosis; and (3) determine risk factors associated with TB development among PHIV in Georgia.

## METHODS

### Study Design

A retrospective cohort study was conducted among persons newly diagnosed with HIV disease in the country of Georgia from 1 January 2019 to 31 December 2020. The follow-up period covered the period from the date of HIV diagnosis to the date of TB diagnosis, death, or 31 December 2022, whichever occurred first.

### Study Settings

Georgia as an upper middle-income Eastern European country located in the Caucasus region with a population of 3.7 million as of early 2023. There is a universal access to the free-of-charge TB and HIV services in the country, provided through the State Programs and major international donor funding (Global Fund to fights AIDS, TB and Malaria) support grants.

HIV care: The provision of HIV care in Georgia is governed by the National HIV/AIDS Treatment and Care Guidelines developed based on the WHO guidelines [23, 24]. Since 2015, ART has been available for all PHIV [25]. Based on WHO and national guidelines, all PHIV are recommended to be screened for TB with a standard clinical algorithm, requiring chest radiography and GeneXpert MTB/RIF at baseline HIV diagnosis evaluation. However, only patients with suspicion of TB are referred to the National Center for Tuberculosis and Lung Diseases (NCTLD) for thorough examinations (including sputum examination, complete blood count, and chest X-ray) and exclusion of active disease. Per the guidelines, all PHIV who do not report any of the WHO-defined symptoms of current cough, fever, weight loss, or night sweats are unlikely to have active TB and should be offered TB preventive treatment, regardless of their CD4 cell count, as part of a comprehensive package of HIV care [23, 26, 27].

TB care: There is a free-of-charge universal access to all TB-related services in Georgia, including diagnosis and treatment of all forms of TB, diagnosis and treatment of other pulmonary pathologies, and surgery for patients with TB. Besides the central facility (NCTLD) in Tbilisi, TB services in the rest of the country are delivered via 9 regional clinics, with 2 of those having inpatient care, and 57 outpatient TB cabinets, predominantly co-located in primary health care facilities. The National Tuberculosis Program (NTP) actively collaborates with the HIV program in the country. HIV testing and counseling is offered for all people newly diagnosed with TB and their contacts. In case of a positive HIV test at the time of TB diagnosis, patients are further referred to HIV service provider facilities for confirmation, CD4 cell count, RNA viral load tests, and relevant treatment initiation.

TPT: TPT care in Georgia is provisioned and managed by the NTP of Georgia. Before implementing new TPT regimens in late 2021, 6 or 9 months of isoniazid was a standard regimen recommended for TPT in Georgia. According to the most recent national TB management guideline (2023, unpublished), the following regimens are recommended for treatment of latent TB in all groups at risk for TB (eg, PHIV, contacts of people with TB, people with immunosuppression): 3 months of weekly isoniazid and rifapentine; 1 month of daily isoniazid and rifapentine; 3 months of daily isoniazid and rifampicin; 4 months of daily rifampicin monotherapy; 6 or 9 months of daily isoniazid monotherapy; and 6 months of daily levofloxacin for contacts of people with fluoroquinolone-susceptible, drug-resistant TB [15].

Despite universal access and comprehensive guidance, there are no standardized approaches for providing TPT among PHIV and related data collection practices across the HIV service provider facilities in Georgia, resulting in major information discrepancies and/or complete lack of relevant data.

### Study Population

Our study evaluated all persons newly diagnosed with HIV from January 2019 to December 2020 at the Infectious Diseases, AIDS and Clinical Immunology Research Center (IDACIRC) in Tbilisi, as well as 3 additional HIV service provider facilities in Kutaisi, Batumi, and Zugdidi. The 4 HIV service provider facilities are central facilities providing HIV diagnostic services across the country, thus covering all regions of Georgia and limiting the likelihood of missing any new diagnoses to a minimum.

### Inclusion/Exclusion Criteria

All people newly diagnosed with HIV in the country of Georgia during 2019–2020 were eligible for inclusion within the study cohort. PHIV aged <18 years were excluded from the study analysis. Those with active TB disease at time of HIV diagnosis were also excluded from the incidence rate analysis.

### Data Collection

The following datasets were used across service provider facilities to collect all study-related data: (1) IDACIRC electronic database comprising all PHIV newly diagnosed during 2019–2020 in Georgia, including demographic, medical, and treatment information and (2) National Tuberculosis Surveillance Database containing information on all notified TB cases (including with positive HIV status) across 2019–2022.

The HIV program database was linked to the National TB Surveillance database of the NCTLD to identify PHIV who were diagnosed with TB from January 2019 to December 2020, inclusive. A unique national identification (ID) number was used as a linking variable and those with no ID indicated were excluded from final study analysis. Information on HIV status, diagnosis, clinical, and demographic data (eg, sex, age, history of TB, concomitant diseases, alcohol use, smoking, injection drug use) were collected from the electronic databases of the selected HIV service provider facilities and NCTLD. The final data were entered into a password-protected electronic database (REDCap) [28], with limited access to principal investigator only.

### Definitions

The diagnosis of TB was defined as bacteriologically or clinically confirmed pulmonary and/or extrapulmonary TB per guidelines of the NTP of Georgia [29]. The incidence of TB was defined as the number of HIV-positive patients with incident active TB disease per 1000 person-years of follow-up.

### Statistical Analysis

A descriptive analysis using frequencies, proportions, measures of central tendency, and variation were used to describe patient demographic and clinical characteristics. Incidence of active TB was calculated within a follow-up period of minimum of 2 years from HIV diagnosis. Person-years were calculated based on period of observation starting from the date of HIV diagnosis and ending on 1 of the following dates: active TB disease diagnosis, death, or completion of follow-up time (31 December 2022), whichever occurred first. The time to TB diagnosis was calculated and cumulative hazard for TB development was assessed using Nelson-Aalen cumulative hazards survival estimate curve. A Cox proportional hazard model was used to calculate crude and adjusted hazards ratios (HR) and 95% confidence intervals (95% CI) for evaluating risk factors associated with TB disease development among PHIV. Multivariable model was adjusted for patient age, sex, smoking status, TPT initiation, CD4 cell count at baseline, and HIV viral load at time of HIV diagnosis. Data were analyzed using the R (version 4.3.1) statistical program.

### Ethics

Study approvals were obtained from the Ethics Committees of the National Center for Disease Control and Public Health of Georgia, the IDACIRC, as well as the NCTLD. A waiver of informed consent was sought considering the retrospective nature of the study and the data to be collected.

## RESULTS

### Baseline Demographic and Clinical Characteristics

A total of 1190 people were registered as having new HIV diagnoses during 2019–2020 within the HIV care system as per the IDACIRC electronic database. Twenty-five newly registered patients did not have a national ID indicated within the HIV program database and thus were excluded from final study analysis.

The median age in the final cohort of 1165 PHIV was 38 (interquartile range [IQR] 30–48) and 76.3% were male. The majority (n = 842, 72.3%) of newly diagnosed patients with HIV were registered in the capital city, Tbilisi, and the rest were distributed across 3 other HIV service provider facilities in Batumi (n = 135, 11.6%), Kutaisi (n = 82, 7.0%), and Zugdidi (n = 60, 5%). The prevalence of comorbidities was based on baseline examination at time of HIV diagnosis were: hepatitis C virus (HCV) infection in 14 (1.2%) cases, hepatitis B virus infection in 7 (0.6%) patients, and active TB disease in 16 (1.3%) patients. Almost half of the patients were tobacco users (n = 511, 43.9%). Heterosexual mode of HIV transmission accounted for the majority of cases (n = 778, 66.8%), whereas homosexual mode of transmission was reported for only 199 (17.1%) patients, and injection drug use for 172 (14.8%) patients. Twenty-nine percent (n = 337) of patients had CD4 cell count lower than 200 μL at time of HIV diagnosis, with the median (IQR) cell count among study cohort being 328 (159–509).

Overall, 1047 (89.9%) patients were started on ART. Median days to ART initiation from HIV diagnosis was 15 (IQR 10–29). Based on available information, only 135 (11.6%) newly diagnosed patients with HIV were started on TB preventive treatment (mean days to TPT initiation, 42.9) along with ART; none of these patients progressed to active TB disease within the study follow-up period (Table 1). Of those on ART without TPT (n = 914), 27 (3%) developed TB, with 5 cases of TB found among those who did not initiate ART. One hundred and six (9.1%) individuals living with HIV died during the study follow-up period.

**Table 1.** TB Incidence Rate by Baseline Characteristics of Newly Diagnosed PHIV in 2019–2020

Characteristics		n	%	TB Rate Per 1000 p-y (95% CI)
Overall study cohort		1165	100	10 (9.6–10.4)
Sex assigned at birth	Male	889	76.3	9.4 (8.9–9.9)
	Female	276	23.7	**12.2** (**11.4–13.0)**
Age, y	≤29	277	23.8	4.9 (3.8–6.0)
	30–39	339	29.1	8.3 (7.4–9.2)
	40–49	309	26.5	**17.5** (**16.8–18.2)**
	≥50	240	20.6	**9.9** (**9.0–10.8)**
	Mean (SD)	39.3 (11.9)		..
	Median (IQR)	38 (30–48)		..
Region	Tbilisi	842	72.3	8.5 (7.9–9.1)
	ROC^a^	277	23.2	**14.7** (**13.9–15.5)**
Comorbidities (at time of HIV diagnosis)	HCV	14	1.2	0
	HBV	7	0.6	0
	TB^b^	16	1.4	..
Tobacco use		511	43.9	8.3 (7.6–9.0)
Mode of HIV transmission	Heterosexual	778	66.8	9.3 (8.7–9.9)
	Homosexual	199	17.1	7.0 (6.0–8.0)
	IDU	172	14.8	**16.0** (**15.1–16.9)**
	Hem. transfusion	4	0.3	0
	Unknown	12	1.0	**42.6** (**40.2–44.9)**
CD4 at time of HIV diagnosis (performed ∼within 2 wk) (missing n = 82 [7%])	≤200	337	29	**28.0** (**27.4–28.6)**
	>200	746	64	3.2 (2.5–3.9)
	Mean (SD)	357.2 (258.9)		..
	Median (IQR)	328 (159–509)		..
HIV viral load at time of HIV diagnosis (copies/mL) (missing n = 350 [30%])	mean (SD)	414 603 (125 679)		..
	median (IQR)	63 746 (12 130–264 000)		..
Received ART	Yes	1047	89.9	9.1 (8.6–9.6)
	No	118	10.1	**22.0** (**20.9–23.1)**
Days to ART initiation from HIV diagnosis	Mean (SD)	49.6 (136.9)		..
	Median (IQR)	15 (10–29)		..
Developed AIDS		367	31.5	**29.1** (**28.6–29.6)**
Days to AIDS from HIV diagnosis	Mean (SD)	78.0 (225.9)		
	Median (IQR)	4 (1–15)		
Days to TB diagnosis from HIV diagnosis	Mean (SD)	200 (332.4)		
	Median (IQR)	38 (8.5–231.8)		
Started on TPT		135	11.6	
Days to TPT initiation from HIV diagnosis	Mean (SD)	42.9 (45.4)		
	Median (IQR)	27 (17–48)		
TPT duration (wk)	Mean (SD)	16.5 (9.4)		
	Median (IQR)	17 (4.6–25.1)		

The bolded values highlight a significant increase in the TB incidence rate in specific sub-groups compared to the overall cohort.

Abbreviations: AIDS, acquired immunodeficiency syndrome; ART, antiretroviral therapy; HBV, hepatitis B virus; HCV, hepatitis C virus; HIV, human immunodeficiency virus; IDU, injection drug use; IQR, interquartile range; SD, standard deviation; TB, tuberculosis; TPT, tuberculosis preventive treatment.

^a^Rest of the country (ROC): Batumi—135 (11.6%), Kutaisi—82 (7.0%), Zugdidi—60 (5%).

^b^Records of active TB disease at time of HIV diagnosis (excluded from TB incidence analysis).

### Incidence of Active TB Disease

Within the overall study PHIV cohort, 32 (2.7%) progressed to active TB over the 3193.7 person-years of follow-up period, with TB incidence rate at 10 cases per 1000 person-years (95% CI, 9.6–10.4). Compared to the overall study cohort, TB incidence rates were found to be higher within several subgroups; specifically, the rate was slightly higher among female patients of our cohort at 12.2 per 1000 person-years (95% CI, 11.4–13.0) and relatively higher among PHIV of 40–49 year age group and those who had injection drug use indicated as the mode of HIV transmission, at 17.5 per 1000 person years (95% CI, 16.8–18.2) and 16 per 1000 person-years (95% CI, 15.1–16.9), respectively. Among those registered outside of the capital of Tbilisi, the rate equaled 14.7 per 1000 person-years (95% CI, 13.9–15.5). Among those not receiving ART, the TB rate was 22 per 1000 person-years (95% CI, 20.9–23.1); those who had a CD4 count of 200 cells/μL or less at the time of HIV diagnosis and those who developed AIDS also had significantly rather higher TB rates at 28 (95% CI, 27.4–28.6) and 29.1 (95% CI, 28.6–29.6) per 1000 person-years, respectively.

**Figure 1.**
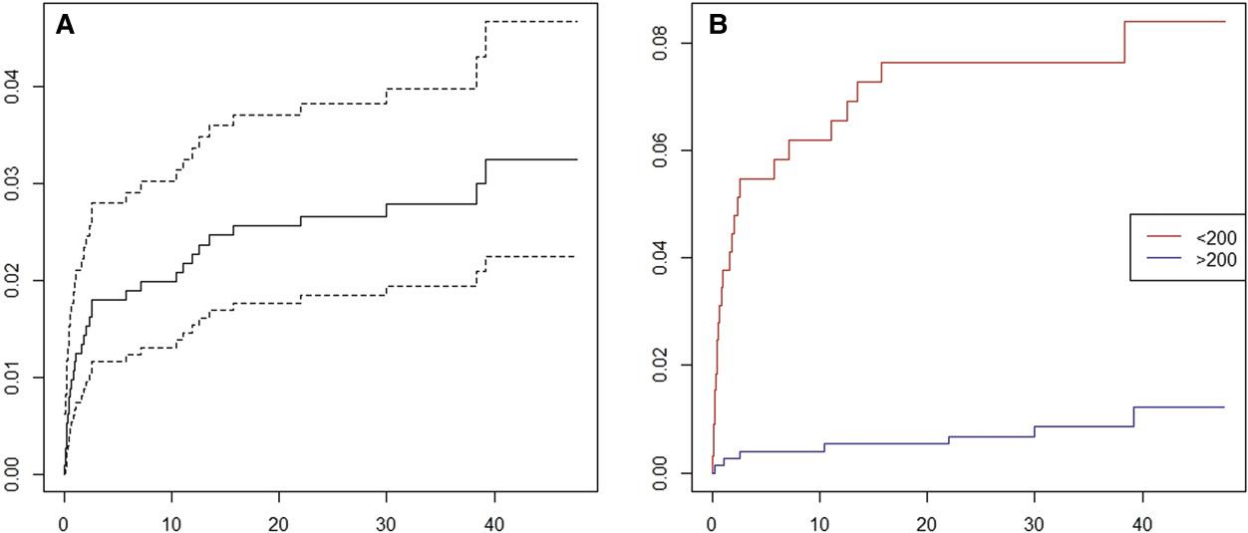
**TB cumulative hazard among people newly diagnosed with HIV during 2019-2020:** (A) TB cumulative hazard estimate over time since HIV diagnosis among PHIV in Georgia—the solid line represents the estimated hazard, whereas the dotted lines indicate the 95% confidence intervals around the estimate; (B) TB cumulative hazard estimate over time since HIV diagnosis by CD4 cell count at time of HIV diagnosis.

The number with developed active TB disease was relatively higher within the first year of HIV diagnosis (mean days = 200, standard deviation = 332.4). The Nelson-Aalen cumulative hazard estimate shows an increase in the risk of getting TB over time; however, there is a sharp increase in the risk of TB within the initial period of less than 5 months from HIV diagnosis (Figure 1). Figure 1B shows the Nelson-Aalen cumulative TB incidence hazard estimate by CD4 cell count, where the risk of developing active TB disease increases steadily over time among those who had CD4 cell count less than 200 μL at the time of HIV diagnosis, compared to those with greater than 200 μL CD4 cell count at baseline (Figure 1).

### Risk Factors for Active TB Disease

An unadjusted regression analysis for risk factors showed a slightly increased risk of developing active TB disease among elder participants (HR, 1.03; 95% CI, 1.01–1.06; *P* value = .03). Higher HIV viral load at baseline (HR, 1.01; 95% CI, 1.001–1.1; *P* value = .001) and having diagnosed AIDS (HR, 12.7; 95% CI, 4.9–32.9; *P* value <.001) were also significantly associated with the development of active TB among PHIV, whereas higher CD4 cell count at baseline was a significant protective factor against developing TB (HR, 0.12; 95% CI; .05–.28, *P* value <.001) (Table 2). Increasing age (aHR, 1.2; 95% CI, 1.03–1.39; *P* value = .01) and AIDS diagnosis (aHR, 3.2; 95% CI, 3.06–27.9; *P* value = .001) were the only risk factors significantly associated with TB development within the final adjusted analysis. A CD4 count >200 cells/μL at the time of HIV diagnosis remained significant as having a protective effect against developing TB (aHR, 0.18; 95% CI, .06–.61; *P* value = .005).

**Table 2.** Cox Proportional Hazards Model for Risk Factors of TB Development Among PHIV

	Unadjusted Analysis			Adjusted Analysis		
Variable	HR	95% CI	*P* value	aHR	95% CI	*P* value
Male	0.77	.36–1.67	.51	1.3	.32–5.49	.68
Age, y	**1.03**	**1.01–1.06**	.**03**	**1**.**2**	**1.03–1.39**	.**01**
Tobacco use	0.85	.34–2.01	.71	0.6	.22–2.12	.51
CD4 cell count >200 at time of HIV diagnosis	**0**.**12**	**.05–.28**	**<**.**001**	**0**.**18**	**.06–.61**	.**005**
Viral load	**1**.**01**	**1.001–1.1**	.**001**	1.009	1.001–1.02	.09
AIDS diagnosis	**12**.**7**	**4.9–32.9**	**<**.**001**	**3**.**2**	**3.06–27.9**	.**001**

The bolded values indicate statistically significant results.

Abbreviations: 95% CI, 95% confidence interval; aHR, adjusted hazard ratio; AIDS, acquired immunodeficiency syndrome; HIV, human immunodeficiency virus; HR, hazard ratio.

## DISCUSSION

To our knowledge, this is a first in-depth study providing insights of the epidemiology and risk factors associated with active TB disease development among people newly diagnosed with HIV in the country of Georgia. We found a relatively high TB incidence rate in our cohort, with a notable concentration of TB incident cases within the first year of HIV diagnosis.

A previous study evaluating TB incidence rate among individuals with both HIV/HCV in Georgia [22] found a higher TB incidence at 14 cases per 1000 person years. This could be potentially explained by overall high TB incidence and high HCV prevalence among Georgian patients with TB at the time of conducting the study, as well as HCV itself being associated with higher TB incidence [30–34]. The rate reported in our study was fairly comparable with that of reported in studies from other countries. A 2014 systematic review and meta-analysis of TB incidence rates among PHIV described a significant heterogeneity between studies, with a summary estimate varying from 4 incident cases per 1000 person-years in cohorts from the low TB burden settings, to 41.7 per 1000 person-years in cohorts from high/intermediate TB burden settings [35]. More recent studies from Uzbekistan and Denmark reported a wide range of TB incident cases within their study populations of PHIV, at 61.2 per 1000 person years (reported 5.1 per 1000 person-months) and 2.9 cases per 1000 person-years, respectively [36, 37]. A 2019 study from Zimbabwe reported 9 TB incident cases per 1000 person years and, similarly to our study, no incident cases were reported among those receiving tuberculosis preventive treatment [38].

In our study, AIDS diagnosis, higher HIV viral load, older age, and lower CD4 cell count at the time of HIV diagnosis were associated with higher risk of developing active TB disease. These results are consistent with previous studies that have highlighted the critical role of immunosuppression, as reflected by lower CD4 cell counts, in predisposing PHIV to developing TB [39–41]. Low CD4 count was also found to be a significant risk factor for TB development among HIV cohorts within number of studies [36, 41, 42]. Our findings contrast with other reports, finding lower age to be significantly associated with increased risk of TB [36], which may be related to overall discrepancies in TB burden settings and other sociodemographic factors. This emphasizes the need of more comprehensive evaluation of related risk factors to further tailor interventions effectively.

A very low prevalence of TB preventive treatment within our cohort indicates that significant barriers exist within the TPT delivery system in Georgia, resulting in high numbers of preventable TB morbidity and mortality among PHIV. Lack of TPT, as well as increased risk of TB within the first year of HIV diagnosis, underscores the importance of timely initiation of TB preventive therapy along with ART, as also evidenced in our study by lack of TB incident cases among those receiving TPT.

Our study has several limitations. First, the retrospective nature of the study may have potentially introduced bias because data collection and data quality assurances processes could not have been accounted for ahead of time. Besides, the lack of information available in the electronic databases have significantly limited the inclusion of certain variables of interest. Future prospective studies could address these limitations and further explore the impact of different sociodemographic and clinical characteristics on the risk of TB development among Georgian PHIV. Second, poor quality of medical records within the HIV care system, resulting in lack of comprehensive clinical information of the PHIV cohort, specifically, status of latent TB infection, status of WHO clinical stage of HIV at time of HIV and/or TB diagnosis, and further information on well-defined and highly reported risk factors of TB across different studies, such as alcohol and/or drug use disorder, diabetes mellitus, undernutrition, and different opportunistic comorbidities [10, 36, 37, 41–44].

The main strength of our study is its uniqueness, which provides valuable insights on the existing situation within the TB/HIV care in Georgia. Within the study, we used nationwide HIV and TB surveillance systems, ensuring all relevant newly diagnosed PHIV were identified for 2 consecutive years and included within the study sample size, as well as all incidences of new TB diagnoses arising from the study PHIV were captured to calculate TB incidence. The study results enhance the overall understanding of TB epidemiology among Georgian PHIV population and emphasizes the need of future prospective and more comprehensive evaluation of related risk factors to further tailor interventions effectively.

The study findings contribute to the growing body of evidence guiding public health strategies aimed at preventing and managing TB among PHIV. Low CD4 cell count and high HIV viral load that were identified as risk factors for active TB in our study underline the importance of ART timely initiation and compliance, along with critically important TB preventive treatment. Nevertheless, it is important to highlight the critical role of prompt diagnosis of HIV infection, which facilitates the timely initiation of ART before the decline of CD4 cell counts below the critical threshold of 200 cells/mm³. Moreover, the study has identified a significantly low intake of TB preventive treatment among Georgian PHIV. Further analysis of the TB diagnostic cascade among people newly diagnosed with HIV in Georgia is needed to evaluate the efficiency, potential gaps, and challenges within the current TPT delivery system in the country to further prevent the development of active TB.

## Notes

***Acknowledgments.*** The authors thank the clinical and administrative staff of the Infectious Diseases, AIDS and Clinical Immunology Research Center and the National Center for Tuberculosis and Lung Diseases in Tbilisi, Georgia, for providing invaluable support and cooperation throughout the study, which was crucial for facilitating data collection and ensuring the smooth execution of quality research activities.

***Author Contributions.*** M.B. and N.T. conceptualized and designed the study. M.B. and D.B. were involved with the development of methodology, data acquisition, analysis, and interpretation. M.B. conducted the literature search and drafted the initial manuscript. All authors critically revised the manuscript and contributed with important intellectual content. M.D. and J.D., the principal recipients of the funding grant, oversaw overall study implementation process. All authors had full access to all the data in the study and had final responsibility for the decision to submit for publication.

***Data Availability.*** All deidentified data will be available on reasoned request from the corresponding author.
